# Schistosomiasis Prevalence and Intensity of Infection in Latin America and the Caribbean Countries, 1942-2014: A Systematic Review in the Context of a Regional Elimination Goal

**DOI:** 10.1371/journal.pntd.0004493

**Published:** 2016-03-23

**Authors:** Ana Clara Zoni, Laura Catalá, Steven K. Ault

**Affiliations:** 1 Independent consultant, Madrid, Spain; 2 Pan American Health Organization/World Health Organization, Washington, D.C., United States of America; University of California Berkeley, UNITED STATES

## Abstract

**Background:**

In 2012 the World Health Assembly adopted resolution WHA65.21 on elimination of schistosomiasis, calling for increased investment in schistosomiasis control and support for countries to initiate elimination programs. This study aims to analyze prevalence and intensity of *Schistosoma mansoni* infection in children in Latin America and the Caribbean countries and territories (LAC), at the second administrative level or lower.

**Methodology:**

A systematic review of schistosomiasis prevalence and intensity of infection was conducted by searching at PubMed, LILACS and EMBASE. Experts on the topic were informally consulted and institutional web pages were reviewed (PAHO/WHO, Ministries of Health). Only SCH infection among children was registered because it can be a ‘proxi-indicator’ of recent transmission by the time the study is conducted.

**Principal Findings:**

One hundred thirty two full-text articles met the inclusion criteria and provided 1,242 prevalence and 199 intensity of infection data points. Most of them were from Brazil (69.7%). Only Brazil published studies after 2001, showing several 'hot spots' with high prevalence. Brazil, Venezuela, Suriname and Saint Lucia need to update the epidemiological status of schistosomiasis to re-design their national programs and target the elimination of *Schistosoma mansoni* transmission by 2020. In Antigua and Barbuda, Dominican Republic, Guadeloupe, Martinique, Montserrat and Puerto Rico schistosomiasis transmission may be interrupted. However the compilation of an elimination dossier and follow-up surveys, per WHO recommendations, are needed to verify that status. Hence, the burden of subtle SCH chronic infection may be still present and even high in countries that may have eliminated transmission. Heterogeneity in the methodologies used for monitoring and evaluating the progress of the schistosomiasis programs was found, making cross-national and chronological comparisons difficult.

**Conclusions:**

There is a need for updating the schistosomiasis status in the historically endemic countries and territories in LAC to address the required public health interventions for control and elimination programs or to verify the elimination of transmission of *Schistosoma mansoni*. Improved reporting and standardization of the monitoring and evaluation methodologies used are recommended, while using available WHO guidelines. Meeting a regional elimination goal will require additional and improved epidemiological data by age group and sex.

## Author Summary

Schistosomiasis (Schistosoma spp) is an intestinal parasitic infection that causes anaemia, stunted growth, impaired cognition, and decreased physical fitness among other pathological effects. Currently, the control of schistosomiasis relies principally on mass drug administration of praziquantel. In addition, the implementation of further interventions such as snail (intermediate host) control, improving access to safe water and sanitation are required to achieve elimination of transmission in humans. An updated epidemiological situation will help countries to design tailor-made interventions enabling a step-up in prevention and control measures with the goal of elimination of transmission of this disease by 2020. In the region of the Americas, *Schistosoma mansoni* is the only species present that infect humans. This systematic review shows that in this region there is a need for re-mapping the schistosomiasis epidemiological situation in certain areas and countries in order to implement and optimize the best public health interventions needed to interrupt transmission or verify elimination of transmission. The methodology for monitoring and evaluating schistosomiasis control programs are well defined by current WHO guidelines. However, further investigations and guidelines on suitable tools for monitoring and evaluating schistosomiasis elimination programs and criteria and procedures for validating the elimination are required, and are currently being undertaken by WHO.

## Introduction

Schistosomiasis (Schistosoma spp) is an infection caused by intestinal and urinary blood fluke parasites. Adult schistosome worms colonize human blood vessels for years,[1] eliminating eggs daily. The chronic infection can cause anemia, stunted growth, impaired cognition, decreased physical fitness, intestinal fibrosis veins, hepatosplenomegaly, neurological complications and death. Subsequently, this disease has a socioeconomic impact on the populations affected.[2,3] The highest prevalence and intensity of infection occur among children 10 to 14 years old.[3,4] Nevertheless, high prevalence and high intensity infections can be detected in pre school-age children (PSAC: 1–4 years old) and persist among adults.[5]

Over 200 million people are estimated to be infected and 700 million people are at risk of schistosomiasis infection globally.[6–8] There are mainly three species [2,9] that can infect humans, but *Schistosoma mansoni* (*S*. *mansoni*) is the only species present in Latin America and Caribbean countries and territories (LAC). In the *S*. *mansoni* endemic areas, the recommendation by WHO for monitoring and evaluation the control of schistosomiasis are the Kato-Katz coprological technique, wherein one can then calculate the arithmetic mean of the eggs per gram of stool (epg) and the severity levels of intensity of infection which are classified as: light (1–99 epg), moderate (100–499) epg, and heavy (500 epg or more).[10,11] Currently, the principle WHO-endorsed public health intervention in schistosomiasis-endemic areas are praziquantel-based (40 mg/kg according to prevalence infection) preventive chemotherapy (PC) depending on the schistosomiasis prevalence: prevalence below 10% once every three years, prevalence between 10% to 49% once every two years and prevalence of 50% or over once a year.[12] This should be accompanied by: snail host control, health education, hygiene promotion, access to safe water and sanitation improvement.[9,13]

In 2001, the World Health Assembly (WHA) set the target of treating with praziquantel at least 75% of school-age children (SAC: 5–14 years old) at risk of schistosomiasis infection by 2010.[14] Even though this target was not attained, in 2012 the WHA recognized the progress made and adopted resolution WHA65.21 on elimination of schistosomiasis, calling for increased investment in schistosomiasis control, and support for countries to initiate elimination programs, where appropriate. [13]. These resolutions are complemented by resolution CD49.R19 of the regional Directing Council of PAHO (2009), calling for a reduction to less than 10% in schistosomiasis prevalence and intensity of infection in high transmission zones by end of 2015.

In LAC, ten countries and territories are considered endemic by WHO. Over 1.6 million people are estimated to require preventive chemotherapy in Brazil and the Bolivarian Republic of Venezuela (hereafter referred to as Venezuela)[15]. Suriname and Saint Lucia may have residual transmission and the six additional countries and territories (Antigua and Barbuda, Guadeloupe, Martinique, Montserrat, Puerto Rico, Dominican Republic) may have eliminated the transmission, but these status need to be verify by compiling an elimination dossier and/or conducting epidemiological surveys.[16]

The objective is to compile data from a set of robust published epidemiological studies, which gives us a view of the prevalence and the intensity of *S*. *mansoni* infection in children in LAC, disaggregated to the second national administrative level (municipality) or lower.

## Methods

A systematic review on schistosomiasis prevalence and intensity of infection among children in LAC was performed. Potentially relevant abstracts were identified in MEDLINE (PubMed), Embase, LILACS (including SciELO) and Cochrane Database of Systematic Reviews. Experts on the topic were consulted informally and institutional web pages were reviewed (PAHO/WHO, Ministries of Health).

The search terms used for research in PUBMED and EMBASE were: ‘‘Schistosomiasis”, ‘‘Child” as MeSH/Emtree terms and a mix of the names of all countries, capitals, and main cities of LAC as text terms. Additional qualifiers were used in PUBMED as MeSH search term “Epidemiology”, “Parasitology” OR “statistics” AND “numerical data”. In LILACS the keywords used were schistosomiasis AND (prevalence OR “intensity of infection”). Detailed information on the search terms and sources is provided online as Supplementary Material.

This review was conducted and reported in accordance with the PRISMA (Preferred Reporting Items for Systematic Reviews and Meta-Analyses) Statement issued in 2009.[17]

The studies included in this review fulfilled the following criteria: (1) Studies published before April 30, 2014; (2) Studies carried out at the second administrative level (municipality) or lower (locality or neighborhood) in countries and territories from LAC; (3) Participants: children (≤19 years old) infected with *S*. *mansoni*; (4) Outcomes: prevalence and/or intensity of infection; (5) Study Design: randomized controlled trials (RCTs), systematic reviews, meta-analyses, cross sectional studies and observational studies. Only SCH infection among children was registered because it can be a ‘proxi-indicator’ of recent transmission by the time the study is conducted.

The excluded studies were those: (1) with sample size less than 30 participants; (2) involving adults that give aggregated results without the possibility of separately analyzing the results in children; (3) involving parasite species but do not report data for *S*. *mansoni;* (4) not published in Portuguese, French, Spanish or English; (5) which calculated prevalence by using clinical diagnosis exclusively; (6) in which the universe is hospital based; (7) with duplicated data.

Two reviewers independently (ACZ and LC) applied the inclusion and exclusion criteria to potential studies, with any disagreements resolved by discussion. For abstracts that met the inclusion criteria the full papers were assessed.

Data were registered based on schistosomiasis prevalence or intensity of infection per location, age groups for children (pre-SAC and SAC) and survey year. Thus, one citation could yield more than one record on the database.

From the extracted information in the database, descriptive analyses of the main findings were developed. Each value of prevalence and intensity of infection registered on the database was denominated a data point. Prevalence was categorized as follows: <1%, ≥1–9%, ≥10–49%, and ≥50%. The intensity of infection was registered as a geometric mean (GM), an arithmetic mean (AM) or unknown (U) when the authors did not report the method used, and the percentage of children by severity levels was also registered according to WHO classification or others.[10,11]

Statistical analysis and mapping were completed using Microsoft Excel, PASW (Predictive Analytics SoftWare) Statistics 18.0 and Tableau 8.2.

## Results

**Fig 1:**
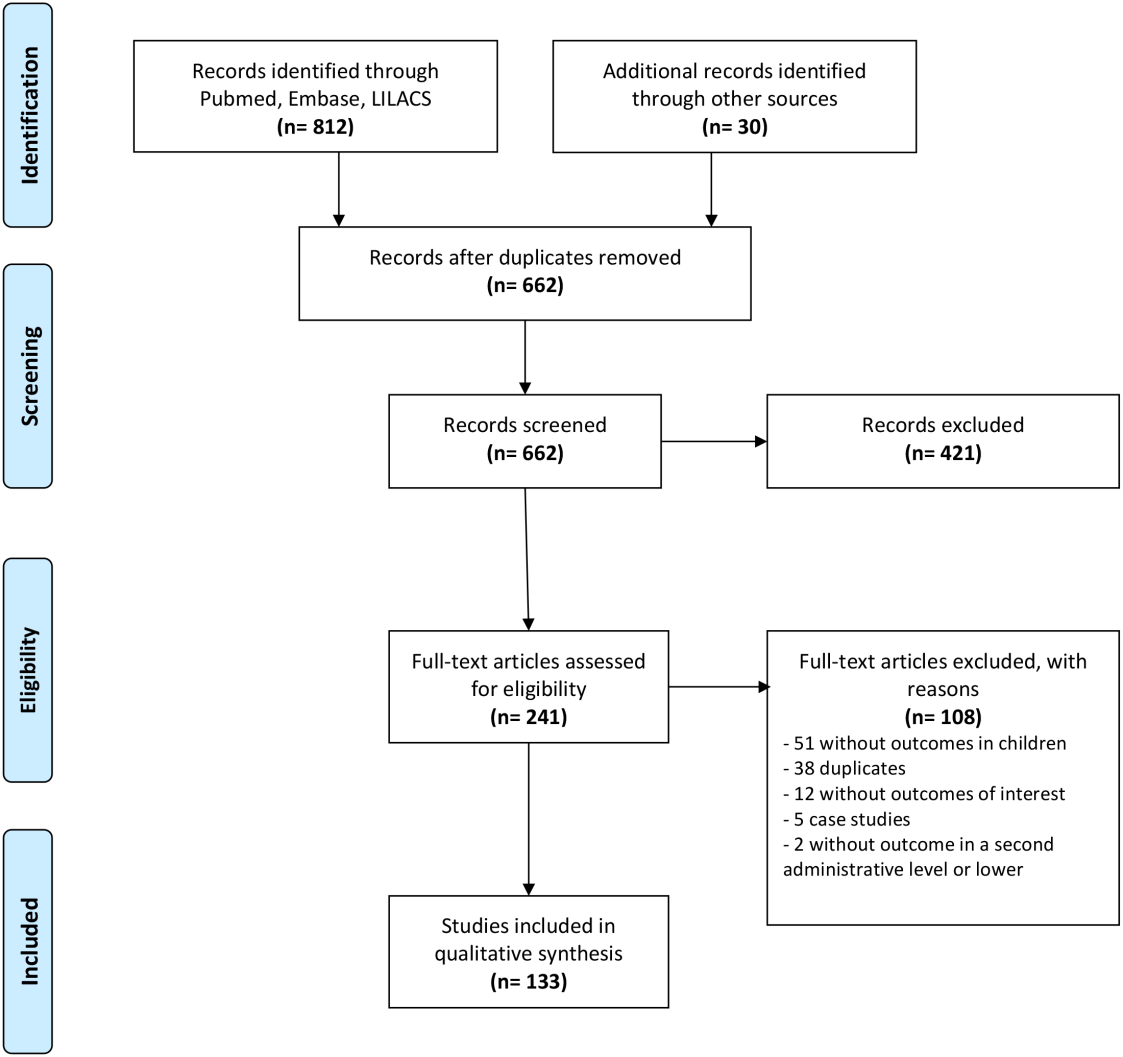
Flow diagram of the systematic review according to PRISMA guidelines.

The initial search identified a total of 842 citations. Most of these studies were excluded because they did not report prevalence or intensity of infection or were cases studies, leaving 241 studies for detailed screening. Of these, 109 were excluded mainly because they did not report outcomes in children. Agreement among the two reviewers was unanimous for the excluded citations. Fig 1 shows the flow diagram of the search, which was organized in accordance with the PRISMA guidelines. One hundred thirty two full-text articles met the inclusion criteria (Table 1) and these provided 1,242 prevalence and 199 intensity of infection data points.

**Table 1:** Studies included in the systematic review and main characteristics.

Author (publication date)	Country	Subarea	Enrollment (year/s or period)	Prevalence % (punctual or range)	Intensity of infection (epg mean, range and severity levels)	Age group (range/s)	Type of test
Amorin et al. (1997)[18]	Brazil	Minas Gerais; 2(2°adm);2(loc)	1997	3.0–57.0	20.0–82.0 (GM)	OC_age (0–9)/ SAC (10–14)	1
Artigas et al. (1969)[19]	Brazil	Sao Paulo; 1(2°adm);1(loc)	1966	0.0–3.4		PSAC (0–5)/ SAC (6–20)	6
Artigas et al.(1970)[20]	Brazil	Sao Paulo; 2(2°adm);2(loc)	1968–1969	0.0–3.8		PSAC (0–5)/ SAC (6–20)	1
Assis et al. (2004)[21]	Brazil	Bahia; 1(2°adm);1(loc)	1992–1993	55.1	WHOc	SAC (7–14)	1
Barbosa et al. (1969)[22]	Brazil	Pernambuco; 1(2°adm);1(loc)	1966/1958-1959	13.6–33.1		PSAC (1–4)/ SAC (5–14)	6
Barbosa et al. (1971)[23]	Brazil	Pernambuco; 1(2°adm);1(loc)	1961/1966	2.4–40.6		PSAC (0–4)/ SAC (5–14)	1
Barbosa et al. (1981)[24]	Brazil	Pernambuco; 1(2°adm);7(loc)	1966/1968/1978	5.8–71.6		PSAC (1–4)/ SAC (5–14)/ Total (1–14)	1
Barbosa et al. (1998)[25]	Brazil	Pernambuco; 1(2°adm);1(loc)	1994	22.6		OC_age (0–9)	1
Barbosa et al. (2000)[26]	Brazil	Pernambuco; 1(2°adm);1(loc)	1977	7.9		SAC	1
Barbosa et al. (2006)[27]	Brazil	Pernambuco; 43(2°adm); 43(loc)	2004	2.6–47.6	23.1–132.4 (GM)	SAC (9–12)	1
Barreto et al. (1984)[28]	Brazil	Bahia; 9(2°adm);9(loc)	1984	0.0–77.9	1.2–55.7 (GM)	SAC (5–14)	1
Barreto et al. (1991)[29]	Brazil	Bahia; 1(2°adm);1(loc)	1984	31.0	93.0 (GM)	SAC (12–14)	1
Bavia et al. (1999)[30]	Brazil	Bahia; 30(2°adm);30(loc)	1990–1993	0.5–61.0		SAC (7–14)	1
Bethony et al. (2002)[31]	Brazil	Minas Gerais; 1(2°adm);1(loc)	1999	30.5–71.8	48.0–211.0 (AM)	OC_age (1–9)/ SAC (10–19)	1
Bina et al. (1974)[32]	Brazil	Bahia; 1(2°adm);1(loc)	1969	10.0–54.0		PSAC (0–4)/ OC_age (0–9)/ SAC (10–14)	6
Brito et al. (2006)[33]	Brazil	Bahia; 1(2°adm);1(loc)	1997	18.9	91.3 (GM)	SAC (7–17)	1
Burlandy-Soares et al. (2003)[34]	Brazil	Sao Paulo; 1(2°adm);1(loc)	1980/1998	1.0–36.0	50.0–110.0 (GM)	PSAC (0–5)/ SAC (5–10)/ SAC (10–15)	5
Camargo-Neves et al. (1998)[35]	Brazil	Sao Paulo; 1(2°adm);1(loc)	1992	0.5		SAC (6–18)	5
Carvalho et al. (1987)[36]	Brazil	Minas Gerais; 2(2°adm);2(loc)	1986/1987	0.0–0.7		SAC (7–14)	1
Carvalho et al. (1994)[37]	Brazil	Minas Gerais; 29(2°adm);29(loc)	1990–1992	0.0		SAC (7–14)	1
Carvalho et al. (1997)[38]	Brazil	Minas Gerais; 31(2°adm);31(loc)	1994–1995	0.2		SAC (7–14)	1
Carvalho et al. (1998)[39]	Brazil	Minas Gerais; 13(2°adm);13(loc)	1988	0.0		SAC (7–14)	1
Cotta et al. (1967)[40]	Brazil	Minas Gerais; 1(2°adm); 52(loc)	1957–1964	0.0–30.2		SAC (7–14)	1
Coura et al. (1984)[41]	Brazil	Minas Gerais; 1(2°adm);1(loc)	1983	10.2–48.0		PSAC (0–5)/ SAC (6–15)	6
Coura et al. (1987)[42]	Brazil	Paraiba; 3(2°adm);3(loc)	1984	2.3–50.0	87.3–245.0 (U)	OC_age (0–9)/ SAC (10–19)	1
Coura et al. (1992)[43]	Brazil	Minas Gerais; 4(2°adm);4(loc)	1973/1976/1977/1979	5.6–77.9	46.0–782.0 (U)	PSAC (0–5)/ SAC (6–10); (11–15)	6
Coura Filho et al. (1992)[44]	Brazil	Minas Gerais; 1(2°adm);1(loc)	1974–1975	13.4–67.5	11.1–64.1 (GM)	PSAC (0–4)/ OC_age (5–9)/ SAC (10–19)	1
Coura Filho et al. (1994)[45]	Brazil	Minas Gerais; 1(2°adm);2(loc)	1991	3.0–42.3		PSAC (1–4)/ SAC (5–19)	1
Coura Filho et al. (1995)[46]	Brazil	Minas Gerais; 1(2°adm);3(loc)	1980	3.2–71.1	24.0–796.4 (GM)	PSAC (0–4)/ SAC (5–14)	1
Coura Filho et al. (1996)[47]	Brazil	Minas Gerais; 1(2°adm);2(loc)	1980/1984/1988/ 1992	6.4–58.9	49.1–365.4 (GM)	Total (0–14)	1
Coura Filho et al. (1998)[48]	Brazil	Minas Gerais; 1(2°adm);1(loc)	1985/1995	0.0–62.5	61.6–134.3(GM)	PSAC (0–4)/ SAC (5–14)	1
Cury et al. (1994)[49]	Brazil	Minas Gerais; 1(2°adm);1(loc)	1992–1993	15.4		SAC (6–14)	1
Cutrim et al. (1998)[50]	Brazil	Maranhao; 3(2°adm);3(loc)	1987/1993	0.0–24.5	25.0–83.0 (GM) and OCII (><100)	Total (0–15)	6
da Costa et al. (1980)[51]	Brazil	Pernambuco; 1(2°adm);9(loc)	1977	17.4–50.1		PSAC (0–4)/ SAC (5–14)	1
da Silva et al. (1997)[52]	Brazil	Maranhao; 1(2°adm);1(loc)	1995	3.4–30.0		PSAC (1–4)/ SAC (5–14)	1
de Lima e Costa et al. (1985)[53]	Brazil	Minas Gerais; 1(2°adm);1(loc)	1974/1981	69.9–70.4	283.3–784.8 (GM)	Total (0–15)	1
de Souza Gomes et al. (2014)[54]	Brazil	Pernambuco; 1(2°adm);1(loc)	2010	20.5		SAC (10–19)	1
de Vlas et al. (1997)[55]	Brazil/ Suriname	Minas Gerais; 1(2°adm);1(loc)/ Saramacca; 1(2°adm);1(loc)	1997	Brazil: 48.9–55.0; Suriname:38.0	Brazil: 99.0–171.0 (GM); Suriname:79.0 (GM)	SAC (11–13); (14–18)/ Total (1–20)	1
Dias et al. (1953)[56]	Brazil	Minas Gerais; 1(2°adm); 1(loc)	1952	3.2–12.4		OC_age (0–10)/ SAC (11–20)	1
Disch et al. (2002)[57]	Brazil	Minas Gerais; 1(2°adm);1(loc)	1991/1996	10.0–55.0		OC_age (0–9)/ SAC (10–14)	1
Enk et al. (2008)[58]	Brazil	Minas Gerais; 1(2°adm);1(loc)	2005–2006	19.7–40.7	58.0 (GM) and WHOc	OC_age (0–10)/ SAC (10–20)	1
Enk et al. (2010)[59]	Brazil	Minas Gerais; 1(2°adm);1(loc)	2004–2006	3.4–20.2	65.1–160.1 (GM)	OC_age (0–10)/ SAC (10–20)	1
Fleming et al. (2006)[60]	Brazil	Minas Gerais; 1(2°adm);1(loc)	2004	35.0–53.0	140.0–180.0 (AM)	OC_age (0–9)/ SAC (10–15)	1
Fontes et al. (2003)[61]	Brazil	Alagoas; 1(2°adm);1(loc)	1999	2.4		SAC (5–18)	1
Fravre et al. (2009)[62]	Brazil	Pernambuco; 1(2°adm);1(loc)	2004	20.5		SAC (7–14)	1
Froes et al. (1970) [63]	Brazil	Sao Paulo; 1(2°adm);1(loc)	1960–1969	0.0–1.3		PSAC (0–4)/ SAC (5–19)	1
Galvao et al. (2010)[64]	Brazil	Pernambuco; 1(2°adm);1(loc)	2006	45.0	335.0 (GM)	SAC (10–19)	1
Gazzinelli et al. (2006)[65]	Brazil	Minas Gerais; 1(2°adm);1(loc)	2001–2002	48.9	68.0 (GM)	OC_age (2–14)	1
Gazzinelli et al. (2006)[66]	Brazil	Minas Gerais; 1(2°adm);1(loc)	2003	77.0	325.0 (U)	SAC (6–18)	1
Goncalves et al. (2005)[67]	Brazil	Rio de Janeiro; 1(2°adm);6(loc)	1996	3.0–18.0		OC_age (1–10)/ SAC (11–20)	5
Grault et al. (1998)[68]	Brazil	Rio de Janeiro; 1(2°adm);4(loc)	1998	2.0		SAC	1
Guimaraes et al. (1985)[69]	Brazil	Minas Gerais; 1(2°adm);1(loc)	1983	32.7	149.7 (GM) and OCII (≤499/500-999/≥1000)	SAC (6–14)	1
Guimaraes et al.(1985)[70]	Brazil	Minas Gerais; 1(2°adm);1(loc)	1981	5.0–55.0	20.0–70.0 (GM)	PSAC (0–4)/ SAC (5–9);(10–14)	1
Guimaraes et al. (2006)[71]	Brazil	Bahia; 1(2°adm);1(loc)	2004	30.2		SAC (6–14)	1
Kanamura et al. (1998)[72]	Brazil	Sao Paulo; 1(2°adm);3(loc)	1991	8.6	57.8 (GM)	SAC (5–18)	5
Kanamura et al. (1998) [73]	Brazil	Sao Paulo; 1(2°adm);2(loc)	1991	0.0–0.7		SAC (5–18)	5
Katz et al. (1983)[74]	Brazil	Minas Gerais; 1(2°adm);1(loc)	1980	0.4		SAC (5–14)	1
Kawazoe et al. (1981)[75]	Brazil	Sao Paulo; 1(2°adm);1(loc)	1979	7.0		SAC (7–13)	8
kloetzel et al. (1987)[76]	Brazil	Pernambuco/Alagoas; 2(2°adm);2(loc)	1956-1957/1963/1976/1983/1984-1985	37.0–94.0	50.0–630.0 (AM) and OCII (><500)	SAC (7–14)	1
kloetzel et al. (1992)[77]	Brazil	Alagoas; 1(2°adm);1(loc)	1991	8.2–53.5	20.0–374.0 AM); 20.0–987.0 (GM) and WHOc	PSAC (2–4)/ SAC (5–14)	1
Lambertucci et al. (1996)[78]	Brazil	Minas Gerais; 1(2°adm);1(loc)	1996	40.0–82.0	90.0–250.0 (AM)	OC_age (0–9)/ SAC (10–19)	5
Lambertucci et al. (2001)[79]	Brazil	Minas Gerais; 1(2°adm);1(loc)	1993–1997	78.0	213.0–298.0 (U)	SAC (10–19)	1
Leal Neto et al. (2012)[80]	Brazil	Pernambuco; 1(2°adm);1(loc)	2008–2009	4.2–34.2		OC_age (0–9)/ SAC (10–19)	1
Lehman et al. (1976)[81]	Brazil	Bahia; 1(2°adm);1(loc)	1970	32.8–75.7	124.0–301.0(GM)	PSAC (1–4)/ SAC (5–14)	1
Lima e Costa et al. (1996)[82]	Brazil	Minas Gerais; 2(2°adm);2(loc)	1987	3.0–4.2		SAC (7–14)	1
Lima et al. (1998)[83]	Brazil	Sao Paulo; 1(2°adm);1(loc)	1992	0.4		SAC (6–18)	5
Marcal et al. (1991)[84]	Brazil	Sao Paulo; 1(2°adm);1(loc)	1987	1.1–7.5	40–75.9 (AM)	PSAC (0–5)/ SAC (5–14)	1
Massara et al. (2004)[85]	Brazil	Minas Gerais; 1(2°adm);3(loc)	2001	8.6	50.7 (GM)	SAC	1
Melo et al. (1983)[86]	Brazil	Maranhao; 17(2°adm);17(loc)	1983	0.0–28.3		SAC (7–14)	1
Mota et al. (1987)[87]	Brazil	Bahia; 1(2°adm);3(loc)	1978	21.1–92.0	10.0–205.0 (GM)	PSAC (1–4)/SAC (5–14)	1
Moza et al. (1998)[88]	Brazil	Pernambuco; 1(2°adm);1(loc)	1994	86.5		Total (2–19)	1
Olliaro et al. (2011)[89]	Brazil	Pernambuco; 1(2°adm);1(loc)	2006	48.0		SAC (10–19)	1
Palmeira et al. (2010)[90]	Brazil	Alagoas; 2(2°adm);2(loc)	2006–2008	20.9–27.7	83.2–187.9 (U)	SAC (7–15)	1
Paraense et al. (1983)[91]	Brazil	Espirito Santo; 36(2°adm);36(loc)	1978–1980	0.0–26.2		SAC (7–14)	1
Paredes et al. (2010)[92]	Brazil	Pernambuco; 1(2°adm);1(loc)	2006–2007	5.0–24.0	OCII: ≤99/100-499/≥500	OC_age (≤9)/ SAC (10–19)	1
Paulini et al. (1971) [93]	Brazil	Minas Gerais; 1(2°adm);1(loc)	1966/1968	7.3–18.5		SAC (7–15)	1
Pereira et al. (2010)[94]	Brazil	Pernambuco; 6(2°adm);19(loc)	1995/1997/2000/2003/2004	0.0–82.1		PSAC (0–5)/ SAC (6–15)	1
Pereira et al. (2010) [95]	Brazil	Bahia; 1(2°adm);1(loc)	2009	44.4		SAC (10–19)	1
Pereira et al. (2010)[96]	Brazil	Minas Gerais; 1(2°adm);1(loc)	2001	64.9	93.2 (GM)	SAC (6–14)	1
Perez et al. (1975)[97]	Brazil	Sao Paulo; 1(2°adm);2(loc)	1972–1974	0.3–1.2		PSAC (1–5)/ SAC (6–20)	1
Pieri et al. (1998)[98]	Brazil	Pernambuco; 1(2°adm);1(loc)	1990	44.1–76.9	129.4–304.8 (GM) and OCII (<100)	OC_age (0–6)/ SAC (7–13)	1
Rocha et al. (2000)[99]	Brazil	Minas Gerais; 1(2°adm);1(loc)	1995	0.0–0.1		OC_age (0–6)/ SAC (7–14)	1
Rodrigues et al. (1995)[100]	Brazil	Minas Gerais; 1(2°adm);1(loc)	1992	3.1–12.4	18.8–464.1 (GM)	PSAC (0–5)/ OC_age (0–10)/ SAC (10–15)	6
Rodrigues et al. (2000)[101]	Brazil	Minas Gerais; 3(2°adm);3(loc)	1984	5.9–20.0		OC_age (0–6)/ SAC (7–14)	1
Santana et al. (1996)[102]	Brazil	Bahia; 1(2°adm);1(loc)	1986	7.7		SAC (7–14)	1
Santana et al. (1997)[103]	Brazil	Bahia; 3(2°adm);3(loc)	1990	16.3–82.7		SAC (7–14)	1
Schall et al. (1993)[104]	Brazil	Minas Gerais; 1(2°adm);1(loc)	1988	12.9		SAC (7–14)	1
Tanabe et al. (1997)[105]	Brazil	Pernambuco; 1(2°adm);4(loc)	1989–1990	53.3–94.6	53.0–250.0 (AM); 28.0–232.0(GM)	PSAC (1–5)/ SAC (6–15)	1
Vasconcelos et al. (2009)[106]	Brazil	Minas Gerais; 1(2°adm);3(loc)	2007	0.0–1.6		PSAC (0–4)/ SAC (5–19)	1
Vinha et al. (1968)[107]	Brazil	Rio Grande do Norte; 23(2°adm); 276(loc)	1965–1966	0.0–100		SAC (7–14)	1
Ximenes et al. (2003)[108]	Brazil	Pernambuco; 1(2°adm);1(loc)	1988	25.2		SAC (10–15)	1
Zacharias et al. (2002)[109]	Brazil	Sao Paulo; 1(2°adm);1(loc)	2000	0.0–31.3		PSAC (1–5)/ SAC (6–14)	2
Hillyer et al. (1983)[110]	Dominican Republic	Hato Mayor; 1(2°adm);1(loc)	1965	31.0		SAC (8–13)	1
Mota et al. (1995)[111]	Dominican Republic	La Altagracia/ Hato Mayor/El Seibo; 3(2°adm);3(loc)	1994	0.0–2.0		SAC (4–14)	1
Read et al. (1966)[112]	Dominican Republic	Hato Mayor; 1(2°adm);1(loc)	1951/1962	21.4–29.2		SAC (6–19)/ (5–14)	1
Vargas M et al. (1987)[113]	Dominican Republic	La Altagracia; 1(2°adm);1(loc)	1984	4.5–12.8	3.3–10.6 (GM)	PSAC (0–4)/ SAC (5–14)	1
WHO (1987)[114]	Dominican Republic	El Seibo/ La Altagracia; 2(2°adm);2(loc)	1972	17.0–27.8		OC_age (3–12)/ SAC (12–20)	3
Lapierre et al. (1972)[115]	Guadeloupe	Point a Pitre; 1(2°adm);1(loc)	1972	4.0		Total (0–15)	2
WHO et al. (1987)[116]	Guadeloupe	Basse-Terre; 6(2°adm);12(loc)	1969–1973	4.0–76.4		SAC	5
WHO et al. (1987)[117]	Martinique	Le Marin/ Saint-Pierre/ Trinite;12(2°adm);12(loc)	1970	0.0–45.0		OC_age (0–10)/ SAC (11–20)	5
Tikasingh et al.(1982)[118]	Montserrat	Saint George; 0(2°adm);2(loc)	1978	0.0–5.0		PSAC (0–4)/ SAC (5–14)	1
Palmer et al. (1969)[119]	Puerto Rico	Patillas/ Caguas; 2(2°adm);2(loc)	1952	12.1–16.4		SAC (6–7)	1
Ferguson et al. (1965)[120]	Puerto Rico	7(1°adm); 8(2°adm);8(loc)	1954/1955/1957/1958	0.3–32.0		SAC (6–10)	1
Ferguson et al. (1968)[121]	Puerto Rico	Vieques/ Caguas; 7(2°adm);7(loc)	1954–1955	1.3–12.4		SAC (6–9)	1
Giboda et al. (1997)[122]	Puerto Rico	Las Piedras/ Corozal/ San Lorenzo; 3(2°adm);3(loc)	1995–1996	0.0		PSAC (1–5)/ SAC (6–15)	1
Hiatt et al. (1978)[123]	Puerto Rico	Las Piedras; 1(2°adm);1(loc)	1972–1974	27.2		Total (0–15)	6
Hiatt et al.(1980)[124]	Puerto Rico	Las Piedras; 1(2°adm);1(loc)	1972–1977	13.3		Total (0–14)	1
Hillyer et al. (1999)[125]	Puerto Rico	19(1°adm); 19(2°adm);19(loc)	1991–1995	0.5–14.0		PSAC (1–5)/ SAC (6–15)	2
Jobin et al. (1968)[126]	Puerto Rico	Aibonito; 1(2°adm);1(loc)	1966	20.5		SAC (6–12)	1
Jobin et al (1970)[127]	Puerto Rico	Guayama/Arroyo/Caguas; 3(2°adm);3(loc)	1953	8.1–16.2		SAC (6)	1
Maldonado et al. (1958)[128]	Puerto Rico	6(1°adm);6(2°adm); 6(loc)	1953	5.3–24.4		PSAC (0–5)/ SAC (5–14)	1
Negron et al. (1978)[129]	Puerto Rico	Juncos; 1(2°adm);1(loc)	1969	27.9		SAC (4–15)	8
Negron et al. (1979)[130]	Puerto Rico	80(1°adm); 80(2°adm);80(loc)	1976	1.0–20.7		SAC (10–13)	3
Negrón et al. (1979)[131]	Puerto Rico	78(1°adm); 78(2°adm);78(loc)	1963	6.0–72.0		SAC (11)	3
Tiben et al.(1973)[132]	Puerto Rico	80(1°adm); 80(2°adm);80(loc)	1969	4.3–27.7		SAC (11)	3
White et al. (1957)[133]	Puerto Rico	16(1°adm); 16(2°adm);16(loc)	1953	0.0–29.9		SAC (5–18);(1–16)	1
Barnish et al. (1982)[134]	Saint Lucia	Region 2; 0(2°adm);1(loc)	1977/1981	0.3–2.3		PSAC (0–5); (0–4)/ SAC (6–14); (5–14)	1
Bartholomew et al. (1981)[135]	Saint Lucia	Region 5; 0(2°adm);1(loc)	1981	9.8–74.2		PSAC (0–4)/ SAC (5–19)	1
Cook et al. (1977)[136]	Saint Lucia	Region 4 y 5; 0(2°adm);2(loc)	1972	6.3–40.2		PSAC (0–4)/ SAC (5–14)	1
Jordan et al. (1975)[137]	Saint Lucia	Region 5; 0(2°adm);5(loc)	1968–1970	18.9–69.1	20.0–72.0 (GM)	PSAC (0–5)/ SAC (6–14)	1
Jordan et al. (1976)[138]	Saint Lucia	Region 2 y 5; 0(2°adm);2(loc)	1971	4.5–37.9		PSAC (0–5)/ SAC (6–14)	1
Jordan et al. (1980)[139]	Saint Lucia	Region 2; 0(2°adm);1(loc)	1975	1.3–9.1		PSAC (0–4)/ SAC (5–14)	1
Jordan et al. (1982)[140]	Saint Lucia	Region 4; 0(2°adm);5(loc)	1973/1980	2.6–23.7		PSAC (0–4)/ SAC (5–14)	1
Jordan et al. (1982)[141]	Saint Lucia	Region 5; 0(2°adm);5(loc)	1975	6.7–35.1	13.0–17.0 (GM)	PSAC (0–4)/ SAC (5–14)	1
Kurup et al. (2010)[142]	Saint Lucia	National administration; 0(2°adm);3(loc)	1996	0.7	78.0 (U)	Total (0–14)	1
Prentice et al. (1981)[143]	Saint Lucia	Region 1; 0(2°adm);5(loc)	1974–1976	43.7		Total (0–14)	1
Prentice et al. (1981)[144]	Saint Lucia	Region 1, 4 y 8; 0(2°adm);3(loc)	1974–1975	29.6–66.4		Total (0–14)	1
Van Der Kuup et al. (1971)[145]	Suriname	Marowijne;1(2°adm);1(loc)	1967	6.6		Total (0–15)	1
Van der Kuyp et al. (1969)[146]	Suriname	Saramacca;3(2°adm);4(loc)	1961–1964	5.6–18.7		PSAC (0–4)/ SAC (5–14)	1
Van Lieshout et al. (1995)[147]	Suriname	Saramacca; 1(2°adm);1(loc)	1995	10.0–30.0		OC_age (0–10)/ SAC (10–19)	9
Alarcón et al. (2007)[148]	Venezuela	Aragua/ Carabobo/ Vargas; 5(2°adm);5(loc)	1998–2000	0.6–6.9		PSAC (0–5)/ SAC (6–15)	5
Scott et al. (1942)[149]	Venezuela	Aragua/ Carabobo/ Miranda/ Distrito Federal/ Vargas; 22(2°adm);39(loc)	1937–1939	0.0–58.0		OC_age (0–10)	1

Epg: eggs per gram of feces

Subarea: Name of the 1^st^ administrative level; number of municipalities or 2^nd^ administrative level; number of localities (cities or villages)

Intensity of infection: AM: Arithmetic mean; GM: Geometric mean; U: Unknown type of mean used; WHOc: WHO classifies severity of infection for *S*. *mansoni as follows*: severe (>400 epg), moderate (100–399 epg) and light (1–99 epg); OCII: report other classification of intensity of infection different from WHO guidelines

Age groups: PSAC: pre-school aged children (≥1–4 years); SAC: school-aged children (≥5–14 years); Total: all children (≥1–14 years); (4) OC_age: Other age group classification.

Type of test: (1) coprologic; (2) serologic; (3) dermatologic; (4) urine test; (5) 1+2; (6) 1+3; (7) 1+4; (8) 1+2+3; (9) 1+2+4

A summary of the main methodological features of the studies included in the descriptive analysis is given in Table 2.

**Table 2:** Descriptive analysis of the main features of the articles included in the systematic review (N = 132).

Variable	Categories	N	%
Countries	Brazil	92	69.7
	Puerto Rico	15	11.2
	Saint Lucia	11	8.2
	Dominican Republic	5	3.7
	Others (Guadeloupe, Suriname, Venezuela, Martinique, Monserrat)	10	8.2
Sample year	1937–1999	111	84.1
	2000–2010	21	15.9
Type of Sample	Universal/Census	20	15.2
	Random	25	18.9
	Did not report the type of sample	87	65.9
Setting	School	46	34.8
	Community	86	65.2
Population area	Rural	75	56.8
	Urban	25	18.9
	Mixed population	27	20.5
	Rural and Urban in the same article	5	3.8
Gender Difference	No	8	6.1
	Yes	39	29.5
	Not analyzed	85	64.4
Age Group	2 groups: PSAC and SAC	36	27.3
	Only SAC (complete group)	16	12.1
	SAC (subgroups)	40	30.3
	SAC (did not report the age)	5	3.8
	Total	12	9.1
	Other classification	23	17.4
Type of test	Stool (fecal)	103	78.0
	Serologic	3	2.3
	Dermatologic	4	3.0
	Urine test	0	0.0
	Combination of above mention	21	15.9
	Did not report any type of test	1	0.8
Intensity of Infection	Arithmetic mean only	4	3.1
	Geometric mean only	25	19.4
	WHO categories only	1	0.8
	Arithmetic mean + Geometric mean	1	0.8
	Arithmetic mean + Geometric mean+ WHO categories	1	0.8
	Arithmetic mean + Other classification	1	0.8
	Geometric mean + WHO categories	1	0.8
	Geometric mean + Other classification	3	2.3
	Other classification	1	0.8
	Unknown type of mean used	6	4.7
	Did not report intensity of infection	85	65.9
Intermediate hosts	*Biomphalaria glabrata*	37	28.0
	*Biomphalaria glabrata* and others	13	9.8
	Others different to *Biomphalaria glabrata*	17	12.9
	Did not report any intermediate hosts	65	49.2

Age group: PSAC: pre-school aged children (≥1–4 years); SAC: school-aged children (≥5–14 years); Total: all children (≥1–14 years)

Studies were identified for 9 of the 45 countries and territories of LAC; 2 from Latin America (Brazil and Venezuela) and 7 from the Caribbean (Dominican Republic, Guadeloupe, Martinique, Montserrat, Puerto Rico, Saint Lucia and Suriname) published between the 1^st^ January 1942 and April 30, 2014. Even though an article from Antigua and Barbuda was identified, it was not included in the systematic review because it did not meet the inclusion criteria. The scientific literature mainly stemmed from Brazil (69.7%).

Most of the studies in the survey were conducted in the community compared to those that were conducted in schools, 65.2% vs. 34.8%, respectively. These were mainly concentrated in rural communities (57.8%). More than half of the studies did not report the sample methodology (66.9%) and did not analyze the results by sex (64.4%).

Sixty seven studies analyzed the intermediate host responsible for the transmission of schistosomiasis infection and the most frequent was the snail *Biomphalaria glabrata* (59.7%).

Heterogeneity in the diagnostic test used was found: 78.0% conducted solely a stool test and 15.9% combined and compared these with serological, dermatological and/or urinary tests. The Kato-Katz technique was the most used (55.6%).

Categorization according to age into two age groups, (PSAC, 1–4 years old and SAC, 5–14 years old) was performed in 36 articles (27.3%). The remaining articles addressed the analysis exclusively in SAC, with children of different ages (46.2%) or without disaggregating in age groups (9.1%), or created other classifications (17.4%).

All the included studies reported prevalence but only 33.3% (44 articles) reported the intensity of infection, being in 4 countries (Brazil, Dominican Republic, Saint Lucia and Suriname). Diversity was also observed in the way various authors quantified intensity of infection: most of the articles used a geometric mean (23.5%), followed by an arithmetic mean (5.3%). Only three articles (2.3%) from Brazil reported the levels of intensity of infection according to the categories of WHO.

Regarding the distribution of the prevalence points for *S*. *mansoni* data by countries, most were recorded for Brazil (727 points) and Puerto Rico (333 points). The remaining seven countries and territories generated a total of 184 prevalence data points.

**Fig 2:**
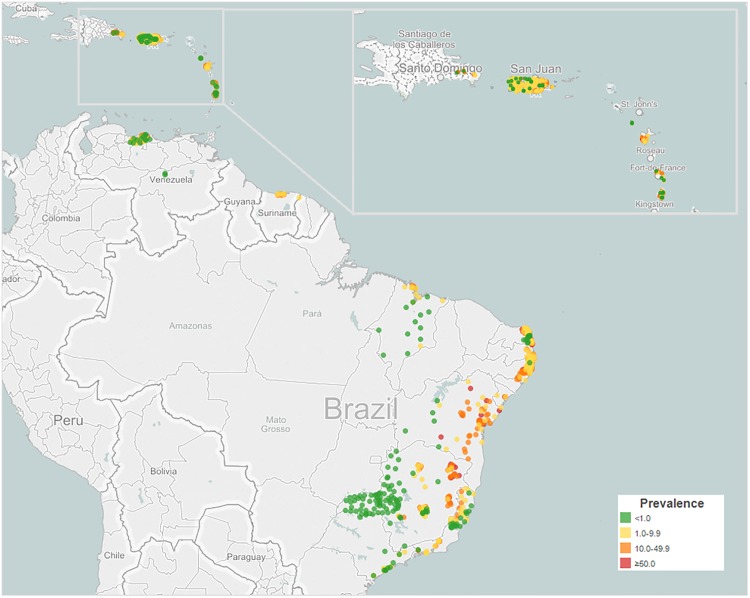
Prevalence of schistosomiasis infection in the Latin America and Caribbean countries and territories, 1942–2014.

In the entire study period the highest prevalence was observed by country as follows: in Brazil, in Minas Gerais for pre-SAC (62.5%) and in Pernambuco for SAC (94.6%); in Puerto Rico with higher values in Luquillo and Rio Grande (72.0% in SAC); in Guadeloupe in Basse-Terre (76.4% in SAC); in Saint Lucia in the region 5 (74.2% in SAC) and in Venezuela in Miranda (58.0%). The highest prevalence in the range of 10–49% were observed in: Martinique in Trinite (45.0% in SAC); in Dominican Republic in Hato Mayor (31.0% in SAC) and in Suriname in Saramacca (38.0% for all age group) and in the range of 1–9% in Montserrat in Saint George (5.0% in SAC). (Fig 2)

Brazil was the only country with epidemiological studies done after 2001, which results were reported by age group for children. When these data were analyzed alone, several 'hot spots' with high prevalence (over 50%) and moderate in the upper limit of this range were observed (40–49%) in the states of Minas Gerais, Bahia and Pernambuco. (Table 3)

**Table 3:** Prevalence of schistosomiasis in children aged 1–15 years, Brazilian states. Epidemiological surveys conducted between 2000 and 2010.

States	Enrollment (year/s or period)	Data points	Prevalence Range (%)
Alagoas	2006–2008	2	20.9–27.7
Bahía	2004	1	30.2
	2009	1	44.4
Minas Gerais	2001	1	64.9
	2001–2002	1	48.9
	2001–2003	3	8.6
	2003	1	77.0
	2004	2	35.0–53.0
	2005–2006	4	3.4–40.7
	2007	6	0.0–1.6
Pernambuco	2000	8	3.4–55.3
	2003	17	0.0–26.3
	2004	46	2.6–48.7
	2006	2	45.0–48.0
	2006–2007	2	5.0–24.0
	2008–2009	2	4.2–34.2
	2010	1	20.5–20.5
São Paulo	2000	2	0.0–31.3
TOTAL	2000–2010	102	0.0–77.0

In relation to the intensity of infection, 153 out of a total of 199 data points were recorded for Brazil, distributed among eight states. Most of the records (73.7%) belonged to Pernambuco and Minas Gerais, and in these states the highest values were 630.0 epg and 796.4 epg, respectively. In three states (Alagoas, Bahia and Minas Gerais) 20–33% of the children were in the severe category of intensity of infection (over 500 epg). In Saint Lucia a total of 43 data points, covering region 5 (78.0 epg) and an area under national administration (13.0 to 72.0 epg) were recorded. In Dominican Republic only 2 data points were recorded in the province of La Altagracia (3.3 and 10.6 epg) and in Suriname 1 data point in the district of Saramacca (79.0 epg).

## Discussion

This systematic review compiles 132 selected articles with 1,242 prevalence and 199 intensity of infection schistosomiasis data points about children in LAC, published between 1942 and 2014. A total of 9 LAC countries and territories identified as historically endemic areas for schistosomiasis were included.

Based on this review, we suggest the epidemiological status of the historically schistosomiasis endemic countries and territories in LAC needs to be updated, since the last published epidemiological surveys available were conducted: in the 1970s for Martinique, Guadeloupe and Montserrat; the 1980s for Dominican Republic and; the 1990s for Puerto Rico, Saint Lucia, Suriname and Venezuela. The only country with updated survey data in the scientific literature was Brazil, where high-prevalence states ('hot spots') were noted as Minas Gerais, Bahia and Pernambuco. The large number of universities and cohorts of MSc/PhD students concentrated in these populous states may explain the huge number of papers published. The unequal distribution of publications per country (and per state, within Brazil) and the outdated survey information also may reflect, in the worst case scenario, a) the difficulty that the countries face in publishing schistosomiasis data in indexed journals; b) the lack of human and economic resources to conduct epidemiological surveys; and/ or c) the lack of political will/interest for undertaking further surveys as the issue of schistosomiasis is often not considered a priority due to more pressing public health problems or the increasing relevance of other vector-borne diseases (e.g.; chikungunya). In the best case scenario, it may be indicative of interruption of transmission. However, also in these countries or foci where interruption of transmission in humans is suspected, epidemiological surveillance is key to prevent a recurrence of real clinical disease, especially in those countries (Guadeloupe and Brazil) where wild animals may have a role in maintaining transmission.[150,151] Furthermore, additional malacological and animal reservoir host studies might be necessary to design required SCH control and elimination strategies, including surveillance.[152,153] Recently, in collaboration with PAHO and/or other stakeholders some Ministers of Health conducted schistosomiasis surveys that are not yet published: Suriname in 2009/2010, Brazil in 2011/2014 and Dominican Republic in 2013. In order to move towards the verification of elimination of the schistosomiasis in humans it is key that these data and other un-published information are made available.

Despite the fact that Brazil has intensified efforts in schistosomiasis control, the results of the published surveys done after 2001 underscore that schistosomiasis continues to be transmitted in “hot spots” of high prevalence; the expansion of PC with praziquantel which Brazil is now undertaking in prioritized municipalities will benefit the affected population. Brazil and Venezuela reported 27,460 people treated with PC in 2012 (mainly in Brazil).[15] This Fig represented only 1.8% of the 1.6 million people estimated to require PC in the Americas. [15] Of the 5 schistosomiasis-endemic WHO regions requiring PC treatment globally, this WHO region with the lowest schistosomiasis PC coverage as reported for 2012, which could be explained by several reasons: (1) countries are not implementing PC strategies (either massive, targeted or focalized administration); (2) the information systems of the countries are not adapted to report individual versus PC mass treatments; and /or (3) health professionals do not record or support PC interventions. Whatever may be the possible reasons, health professionals should be aware that the public and individual health benefits of PC greatly outweigh the minimal risks which could arise.[12]

Only 44 articles, those from Brazil, Dominican Republic, Saint Lucia and Suriname, out of a total of 132 articles measured intensity of infection. Brazil was the country with the highest number of records of intensity of infection (153; 76.9%) and the only one which reported the percentage of children infected according to WHO’s intensity of infection classification levels.[10,11] The intensity of infection is one of the first indicators which is reduced when schistosomiasis PC is implemented. Therefore, it is very important to monitor the intensity of infection, because when intensity of infection is high it takes more time to reduce the prevalence and the post-treatment reinfections are more frequent. The intensity of infection was expressed as an arithmetic mean, as recommended by WHO, in only 9 articles; the geometric mean was the measure most used (31 articles), despite the fact that it can under- or overestimate the efficacy of praziquantel.[11] The classification by level of intensity of infection allows a quick assessment of the proportion of people who suffer from the serious consequences of this disease and therefore the burden of the disease in the community. Given the above, the draft guidelines for verification of elimination of schistosomiasis as proposed by WHO to control morbidity and to eliminate schistosomiasis as a public health problem established the goals to reach prevalence of severe intensity of infection of less than 5% and 1%, respectively. Therefore, schistosomiasis endemic countries need to assess the prevalence, but also measure intensity of infection, as recommended by WHO.[10]

This systematic review is focused on children to identify recent SCH transmission by the time the included studies were conducted. Therefore, this study may underestimate the prevalence of chronic schistosomiasis infection where transmission might be interrupted years ago but remains in adults and where results were not reported disaggregated by age group. For example, we have not included any data of the western focus in Venezuela (Chabasquen), which is in the confluence of Lara, Portuguesa and Trujillo states. This is an old focus still active, according to the Ministry of Health of Venezuela. [151]

The methodology for monitoring and evaluating schistosomiasis control programs are well defined by current WHO guidelines. However, further investigations and guidelines on suitable tools for monitoring and evaluating schistosomiasis elimination programs and criteria and procedures for validating the elimination of transmissions need to be published by WHO.

Even though there was a large variation in the ages of those surveyed and ways to classify age cohorts, SAC were the most analysed age group. This may be due to the following factors: (1) historically SAC have had the highest rates of SCH infection compared to PSAC or adults; (2) it is more difficult to survey pre-SAC than SAC; (3) the resolution WHA54.19 was aimed at minimum treatment for SAC but not for other age cohorts or groups; and/or (4) praziquantel was not available in paediatric solutions.[154] Nevertheless, new evidence in Africa reports rates in PSAC as high as those of SAC, thus the need for tackling schistosomiasis among this age group, as well.[155,156]

In conclusion, heterogeneity was detected in the methodologies used for the surveys and the way in which results were reported. Therefore, for future studies that attempt to update the epidemiological status, it is recommended that the following methodological suggestions be applied: (1) perform the analysis on children with a description of the results separate from the adult population because the absence of infections in this age group means interruption of transmission; (2) classify all children based on pre-SAC and SAC groups; (3) report the sample size; (4) describe whether the survey was conducted in the entire locality, and if not, what type of sampling was used; (5) specify the diagnostic test used, and if possible use the one recommended by WHO (Kato-Katz stool examination for schistosomiasis control programs); (6) analyze intensity of infection by an arithmetic mean; (7) report the percentage of children infected according to WHO’s intensity of infection classification levels. Additionally, given the scarce gender data on schistosomiasis infection, gender data should be collected.

To reach the elimination goal in the region of the Americas by 2020, there is need for updating the epidemiological status of some of the less-studied states in Brazil and in Venezuela, Suriname and Saint Lucia to address the required public health interventions, such as PC, snail control, improve access to safe water and sanitation, and promote health education. In the remaining countries and territories, evidence must be compiled to see whether interruption of schistosomiasis transmission has occurred.

## Supporting Information

S1 Table Search terms included in databases. (DOCX)

S1 Text Key papers. (DOCX)

S2 Text Learning points. (DOCX)
