# Supplementary material for: Schistosomiasis Prevalence and Intensity of Infection in Latin America and the Caribbean Countries, 1942-2014: A Systematic Review in the Context of a Regional Elimination Goal
Source: PLoS Negl Trop Dis. 2016 Mar 23;10(3):e0004493. doi: 10.1371/journal.pntd.0004493 (PMC4805296; doi:10.1371/journal.pntd.0004493)
Supplement: S1 Table — (DOCX) [file pntd.0004493.s001.docx]

**S1 table. Search terms included in databases**

| **Data sources** | **Key words** |
| --- | --- |
| PUBMED | (((("Schistosomiasis"[Mesh] **OR** ("Schistosomiasis /epidemiology"[Mesh] OR " Schistosomiasis /parasitology"[Mesh] **OR** " Schistosomiasis /statistics and numerical data"[Mesh]))) **AND** ("Child"[Mesh] **OR** ("Child/epidemiology"[Mesh] OR "Child/statistics and numerical data"[Mesh])))) **AND** (“Latin America*” OR “South America” OR “Central America” OR Caribbean OR Anguilla OR “Antigua and Barbuda” OR Argentina OR Aruba OR Bahamas OR Barbados OR Belize OR Bermuda OR Bolivia* OR Brazil* OR “British Virgin Islands” OR “Cayman Islands” OR Chile* OR Colombia* OR “Costa Rica” OR Cuba* OR Dominica OR “Dominican Republic” OR “El Salvador” OR Ecuador OR “French Guiana” OR Grenada OR Guadalupe OR Guatemala OR Guyana OR Haiti OR Honduras OR Jamaica* OR Martinique OR Mexico OR Montserrat OR “Netherlands Antilles” OR Nicaragua* OR Panama OR Paraguay* OR Peru OR “Puerto Rico” OR “Saint Kitts and Nevis” OR “Saint Lucia” OR “Saint Vincent and the Grenadines” OR Suriname OR “Trinidad and Tobago” OR “Turks and Caicos Islands” OR Uruguay* OR Venezuela OR brasil* OR Argentinean OR Mexican OR costaric* OR “the Valley” OR “Saint John's” OR “Buenos Aires” OR Basseterre OR Basse-Terre OR Hamilton OR Oranjestad OR Nassau OR Bridgetown OR Belmopan OR Sucre OR “La Paz” OR Brasilia OR “Sao Paulo” OR “Rio de Janeiro” OR “Belo Horizonte” OR Maceió OR Manaus OR Goiânia OR Belem OR “Porto Alegre” OR Florianopolis OR “George Town” OR Santiago OR Valparaiso OR Concepción OR Temuco OR Bogota OR Cali OR Medellin OR Barranquilla OR “San Jose” OR Havana OR Habana OR “Santo Domingo” OR Roseau OR “San Salvador” OR Quito OR Guayaquil OR “Saint George's” OR “Port au Prince” OR “Port of Spain” OR Tegucigalpa OR Kingston OR Kingstown OR Willemstad OR Managua OR Asuncion OR Lima OR “San Juan” OR Marigot OR Castries OR Paramaribo OR Montevideo OR Caracas OR “Road Town” OR Mexicali OR “Tuxtla Gutiérrez” OR Chihuahua OR Guanajuato OR Guadalajara OR Toluca OR Morelia OR Monterrey OR Puebla) **Filters: Humans** |
| EMBASE | #1 'schistosomiasis'/mj  #2 'child'/de  #3 'south and central america'/exp OR 'south and central america'  #4 'south america'/exp OR 'south america'  #5 'central america'/exp OR 'central america'  #6 'latin america'/exp OR 'latin america'  #7 'latin american'  #8 'caribbean islands'/exp OR 'caribbean islands'  #9 'caribbean'/exp OR 'caribbean'  #10 'african caribbean'/exp OR 'african caribbean'  #11 'anguilla (country)'/exp OR 'anguilla (country)'  #12 'antigua and barbuda'/exp OR 'antigua and barbuda'  #13 puebla  #14 monterrey  #15 morelia  #16 toluca  #17 'guadeloupe'/exp OR 'guadeloupe'  #18 guadalajara  #19 guanajuato  #20 chihuahua  #21 'tuxtla gutierrez'  #22 mexicali  #23 'road town'  #24 caracas  #25 montevideo  #26 paramaribo  #27 castries  #28marigot  #29'san juan'  #30 lima  #31 'aruba'/exp OR 'aruba'  #32 'argentina'/exp OR 'argentina'  #33 asuncion  #34 managua  #35 willemstad  #36 kingstown  #37 kingston  #38 tegucigalpa  #39 'port of spain'  #40 'port au prince'  #41 'saint georges'  #42 guayaquil  #43 quito  #44 'san salvador'  #45 roseau  #46 'santo domingo'  #47 habana  #48 havana  #49 'san jose'  #50 barranquilla  #51 medellin  #52 cali  #53 bogota  #54 temuco  #55 concepcion  #56 valparaiso  #57 santiago  #58 'george town'  #59 florianopolis  #60 'porto alegre'  #61 belem  #62 goiania  #63 manaus  #64 maceio  #65 'belo horizonte'  #66 'rio de janeiro'  #67 'sao paulo'  #68 brasilia  #69 'la paz'  #70 sucre  #71 belompan  #72 bridgetown  #73 nassau  #74 oranjestad  #75 hamilton  #76 'basse terre'  #77 basseterre  #78 'buenos aires'  #79 'saint johns'  #80 'the valley'  #81 costaric*  #82 'mexican'  #83 argentinean  #84 brasil*  #85 'venezuela'/exp OR 'venezuela'  #86 uruguay*  #87 'uruguay'/exp OR 'uruguay'  #88 'turks and caicos islands'/exp OR 'turks and caicos islands'  #89 'trinidad and tobago'/exp OR 'trinidad and tobago'  #90 'suriname'/exp OR 'suriname'  #91 'saint vincent and the grenadines'/exp OR 'saint vincent and the grenadines'  #92 'saint lucia'/exp OR 'saint lucia'  #93 'saint kitts and nevis'/exp OR 'saint kitts and nevis'  #94 'puerto rico'/exp OR 'puerto rico'  #95 'peru'/exp OR 'peru'  #96 paraguay*  #97 'paraguay'/exp OR 'paraguay'  #98 'panama'/exp OR 'panama'  #99 nicaragua*  #100 'nicaragua'/exp OR 'nicaragua'  #101 'netherlands antilles'/exp OR 'netherlands antilles'  #102 'montserrat'/exp OR 'montserrat'  #103 'mexico'/exp OR 'mexico'  #104 'martinique'/exp OR 'martinique'  #105 jamaica*  #106'jamaica'/exp OR 'jamaica'  #107 'honduras'/exp OR 'honduras'  #108'haiti'/exp OR 'haiti'  #109'guyana'/exp OR 'guyana'  #110'guatemala'/exp OR 'guatemala'  #111 guadalupe  #112 'grenada'/exp OR 'grenada'  #113'french guiana'/exp OR 'french guiana'  #114'ecuador'/exp OR ecuador  #115'el salvador'/exp OR 'el salvador'  #116 'dominican republic'/exp OR 'dominican republic'  #117 'dominica'/exp OR 'dominica'  #118 cuba*  #119'cuba'/exp OR 'cuba'  #120'costa rica'/exp OR 'costa rica'  #121 colombia*  #122 'colombia'/exp OR 'colombia'  #123 chile*  #124 'chile'/exp OR 'chile'  #125 'cayman islands'/exp OR 'cayman islands'  #126 'british virgin islands'/exp OR 'british virgin islands'  #127 brazil*  #128 'brazil'/exp OR 'brazil'  #129 bolivia*  #130 'bolivia'/exp OR 'bolivia'  #131 'bermuda'/exp OR 'bermuda'  #132 'belize'/exp OR 'belize'  #133 'barbados'/exp OR 'barbados'  #134 'bahamas'/exp OR 'bahamas'  #135 #3 OR #4 OR #5 OR #6 OR #7 OR #8 OR #9 OR #10 OR #11 OR #12 OR #13 OR #14 OR #15 OR #16 OR #17 OR #18 OR #19 OR #20 OR #21 OR #22 OR #23 OR #24 OR #25 OR #26 OR #27 OR #28 OR #29 OR #30 OR #31 OR #32 OR #33 OR #34 OR #35 OR #36 OR #37 OR #38 OR #39 OR #40 OR #41 OR #42 OR #43 OR #44 OR #45 OR #46 OR #47 OR #48 OR #49 OR #50 OR #51 OR #52 OR #53 OR #54 OR #55 OR #56 OR #57 OR #58 OR #59 OR #60 OR #61 OR #62 OR #63 OR #64 OR #65 OR #66 OR #67 OR #68 OR #69 OR #70 OR #71 OR #72 OR #73 OR #74 OR #75 OR #76 OR #77 OR #78 OR #79 OR #80 OR #81 OR #82 OR #83 OR #84 OR #85 OR #86 OR #87 OR #88 OR #89 OR #90 OR #91 OR #92 OR #93 OR #94 OR #95 OR #96 OR #97 OR #98 OR #99 OR #100 OR #101 OR #102 OR #103 OR #104 OR #105 OR #106 OR #107 OR #108 OR #109 OR #110 OR #111 OR #112 OR #113 OR #114 OR #115 OR #116 OR #117 OR #118 OR #119 OR #120 OR #121 OR #122 OR #123 OR #124 OR #125 OR #126 OR #127 OR #128 OR #129 OR #130 OR #131 OR #132 OR #133 OR #134  #136 **#1 AND #2 AND #135 #137 #136 AND 'human'/de** |
