# Supplementary material for: Schistosomiasis Prevalence and Intensity of Infection in Latin America and the Caribbean Countries, 1942-2014: A Systematic Review in the Context of a Regional Elimination Goal
Source: PLoS Negl Trop Dis. 2016 Mar 23;10(3):e0004493. doi: 10.1371/journal.pntd.0004493 (PMC4805296; doi:10.1371/journal.pntd.0004493)
Supplement: S1 Text — (DOCX) [file pntd.0004493.s002.docx]

**Key papers**

1. Colley DG, Bustinduy AL, Secor WE, King CH (2014) Human schistosomiasis. Lancet 383: 2253-2264. S0140-6736(13)61949-2 [pii];10.1016/S0140-6736(13)61949-2 [doi].
2. World Health Organization (2011) Helminth control in school age children: a guide for managers of control programmes.
3. World Health Organization (2014) Schistosomiasis: number of people receiving preventive chemotherapy in 2012. Wkly Epidemiol Rec 89: 21-28.
4. Olliaro PL, Vaillant MT, Belizario VJ, Lwambo NJ, Ouldabdallahi M, Pieri OS, et al. (2011) A multicentre randomized controlled trial of the efficacy and safety of single-dose praziquantel at 40 mg/kg vs. 60 mg/kg for treating intestinal schistosomiasis in the Philippines, Mauritania, Tanzania and Brazil. PLoS Negl Trop Dis 5: e1165. 10.1371/journal.pntd.0001165 [doi];PNTD-D-10-00083 [pii].
5. Stothard JR, Sousa-Figueiredo JC, Betson M, Green HK, Seto EY, Garba A, et al. (2011) Closing the praziquantel treatment gap: new steps in epidemiological monitoring and control of schistosomiasis in African infants and preschool-aged children. Parasitology 138: 1593-1606. S0031182011001235 [pii];10.1017/S0031182011001235 [doi].
