# Supplementary material for: Schistosomiasis Prevalence and Intensity of Infection in Latin America and the Caribbean Countries, 1942-2014: A Systematic Review in the Context of a Regional Elimination Goal
Source: PLoS Negl Trop Dis. 2016 Mar 23;10(3):e0004493. doi: 10.1371/journal.pntd.0004493 (PMC4805296; doi:10.1371/journal.pntd.0004493)
Supplement: S2 Text — (DOCX) [file pntd.0004493.s003.docx]

**Learning points**

1. Several historically schistosomiasis-endemic countries and territories of the Region of the Americas may have eliminated the transmission of schistosomiasis. Nevertheless, they may still have people with chronic schistosomiasis infections who were infected in the past. There is a need to verify this through epidemiological surveys.
2. The epidemiological status of several countries and territories of the Latin America and Caribbean countries should be updated, supported by improved and standardized methods and tools to monitor and evaluate control and verify elimination of transmission of schistosomiasis.
3. Brazil has some foci where the transmission of schistosomiasis apparently has remained high. The historically SCH-endemic countries in the Americas have information gaps to fill and/or update on geographic distribution, prevalence and intensity of infection in children.
4. Future epidemiological surveys of schistosomiasis in countries in the region should include all pertinent geographic areas and not be limited to areas of greater survey capacity or greater geographic access.
5. The Americas region has the potential to become the first one to reach regional (region-wide) elimination of transmission, perhaps by 2020, but additional key data are needed.
